# Supplementary material for: An All-Solid-State Coaxial Structural Battery Using Sodium-Based Electrolyte
Source: Molecules. 2021 Aug 28;26(17):5226. doi: 10.3390/molecules26175226 (PMC8434136; doi:10.3390/molecules26175226)
Supplement: Supplementary file 1 [file molecules-26-05226-s001.zip › Molecules_Support_Info_final_27082021.pdf]

Support Information

An all-solid-state coaxial structural battery using sodium-based electrolyte

Federico Danzi[a,b*], Pedro Ponces Camanho[b,c], and Maria Helena Braga[a,b*]

[a.]LAETA, Engineering Physics Department, Engineering Faculty, University of Porto, Rua Dr. Roberto Frias, s/n, 4200-465, Porto, Portugal.
[b.]INEGI, Institute of Science and Innovation in Mechanical and Industrial Engineering, Rua Dr. Roberto Frias, 400, 4200-465, Porto, Portugal.
[c.]LAETA, Mechanical Engineering Department, Engineering Faculty, University of Porto, Rua Dr. Roberto Frias, s/n, 4200-465, Porto, Portugal. Email: pcamanho@fe.up.pt
*Correspondence to: fdanzi@inegi.up.pt (F.D.), mbraga@fe.up.pt (M.H.B.)

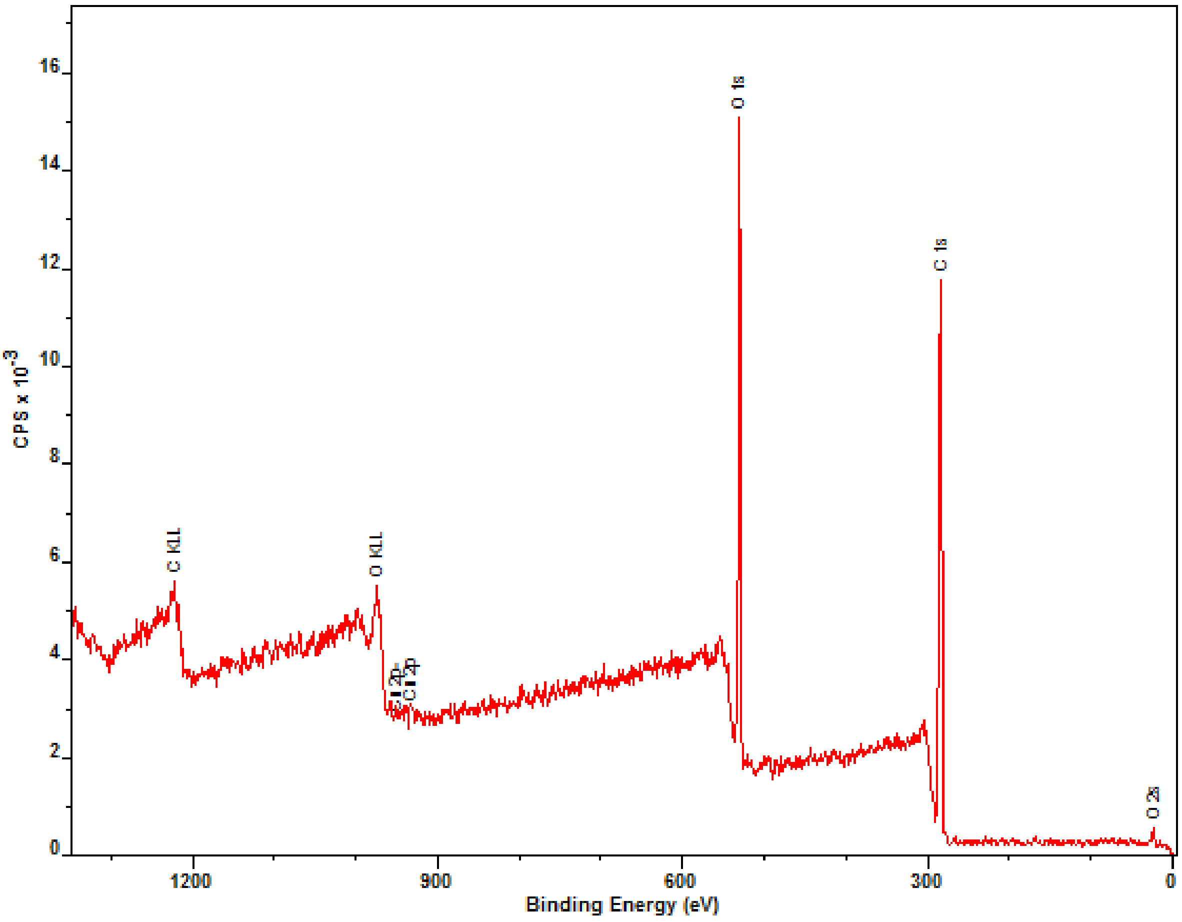

Figure S1. Polyvinyl acetate (PVAc – $C_4H_6O_2$) composite on Cu subtract. XPS survey in full spectra.

Table S1. The atomic percentage of the elements constituting the polymer used to prepare a composite with the ferroelectric Na-glass electrolyte constituted essentially by polyvinyl acetate (PVAc – $C_4H_6O_2$) on Cu substrate (the polymer in this analysis contained no Na-glass).

Analysis of ROI (normalized to 100%)

Element	Sensitivity Factor	At % Pol_Cu
C 1s	0.278	68.8
O 1s	0.78	30.9
Cu 2p3	3.547	0.3

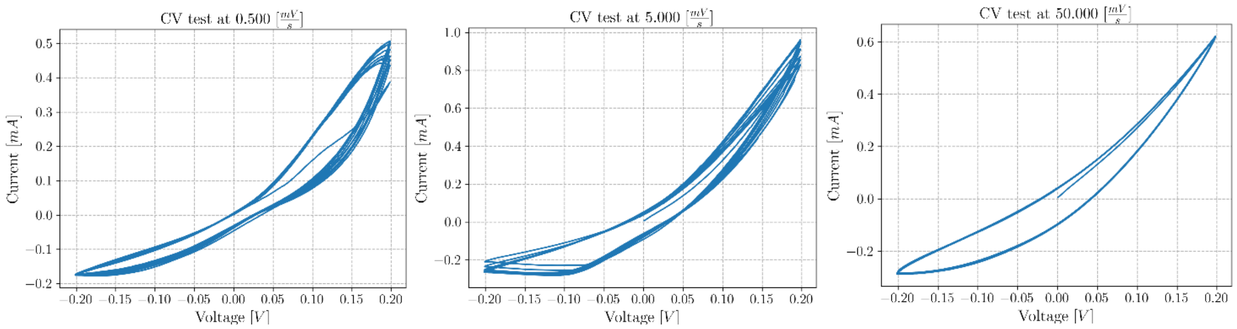

(a) **(b)** **(c)**

Figure S2. Typical cyclic voltammetry curves for a coaxial structural battery at different scan-rates at 40°C (OCV set to 0 V); (a) 0.5 mV/s, (b) 5.0 mV/s and 50 mV/s.

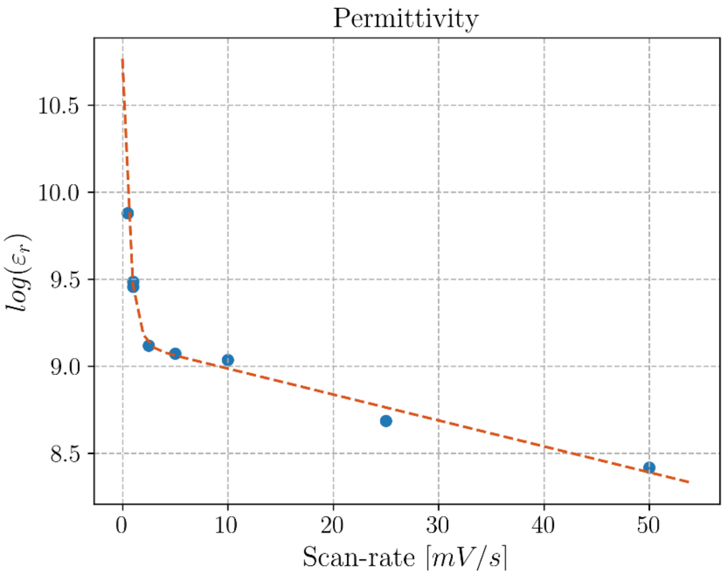

Figure S3. All-solid-state structural coaxial battery cell permittivity was obtained by cyclic voltammetry for different scan-rates at 40 °C with two-phase exponential decay curve fitting.

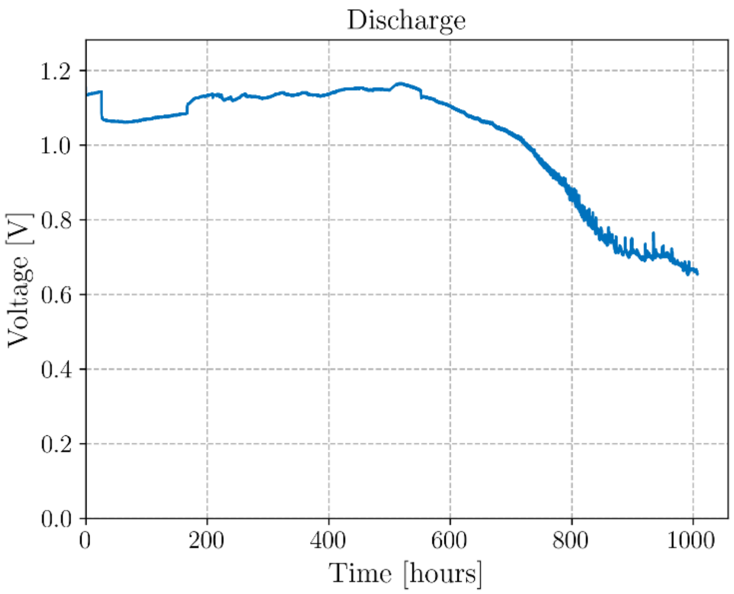

Figure S4. Discharge curves of all-solid-state structural coaxial batteries with 1.8 kΩ material resistors at 40°C (the external resistor was connected to the cell after 25 h).

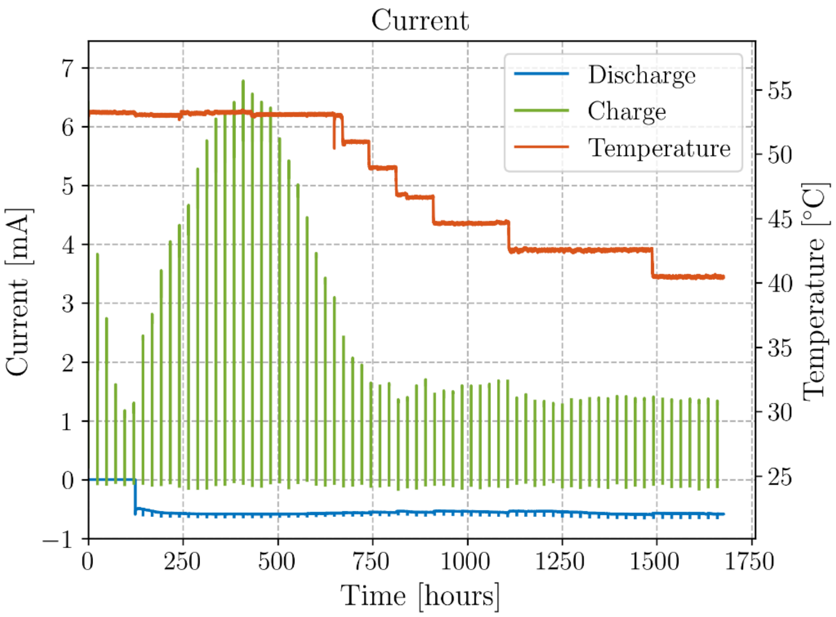

Figure S5. Charge and discharge currents corresponding to the flexural bending post-mortem electrochemical cycling in Figure 9 in which charge is performed at a constant voltage of 1.3 V for 3 min and discharge performed with a material resistor of 1.8 kΩ for 24 h. The temperature varied from ~53 to 40°C.
